# T and NK Cell-Based Immunotherapy in Chronic Viral Hepatitis and Hepatocellular Carcinoma

**DOI:** 10.3390/cells11020180

**Published:** 2022-01-06

**Authors:** Paola Fisicaro, Carolina Boni

**Affiliations:** 1 Laboratory of Viral Immunopathology, Unit of Infectious Diseases and Hepatology, Azienda Ospedaliero-Universitaria di Parma, 43126 Parma, Italy; 2 Department of Medicine and Surgery, University of Parma, 43126 Parma, Italy

In chronic viral hepatitis and in hepatocarcinoma (HCC), antigen-specific T cells are deeply exhausted, and evidence of dysfunction has also been observed for NK cells, which can play a pathogenetic role, exerting a regulatory activity on adaptive immune responses.

Among different described inhibitory mechanisms contributing to T and NK cell impairment, there is the up-regulation of inhibitory receptors, transcriptional, metabolic, and epigenetic deregulation, and nutrient depletion in the hepatic microenvironment.

Immunotherapeutic approaches are widely believed to represent potential therapeutic strategies, and new formulations are being tested for clinical translation.

This Special Issue highlights the present knowledge about the emerging cell-based immune-therapeutic strategies for chronic viral hepatitis and HCC.

Three research articles explore NK cell features in the context of HCC, as a potential target of immunomodulation, while five reviews deal with NK/T cell based immunotherapeutic strategies for HCC or chronic viral hepatitis B and C. A further interesting approach to T cell therapy for liver transplanted HCC patients, based on engineered T cells, is elucidated in the commentary by Bertoletti’s group.

A comprehensive phenotypic analysis of 32 molecules’ expression, including Siglecs and immune checkpoints, in peripheral and intrahepatic NK cells from HCC patients is described by Yoshida et al., with the aim to identify signature molecules expressed by circulating NK cells, correlating with cellular functions and hopefully with the patients’ clinical status to be used as biomarkers. In this study, CD160 and CD49a expression by CD56dim NK cells were shown to represent surrogate markers of activating and inhibitory phenotypes, while Siglec-10 correlated with tumor size [1].

Overexpression of CD49a on HCC-infiltrating NK cells has also been observed in the study by Zecca et al., where an enriched subset of NK cells with a pro-angiogenic profile and reduced cytotoxic potential has been described playing a regulatory role and supporting tumor growth, thus potentially representing a novel target for immunomodulatory therapies [2].

Tumor-infiltrating NK cells have been addressed also in the study by Rennert and colleagues, which analyzes patients with HCC either associated with chronic hepatitis B or with liver cirrhosis. The authors find the HCMV-associated FcɛRIɣ negative adaptive NK cell subset, displaying limited antitumoral activity against liver cancer cells as an intrinsic characteristic conserved among patients and healthy controls, significantly expanded in HBV-associated HCC patients, thus linking the NK cell repertoire to HCC etiology [3].

Generally, the observation, common to HCC and chronic viral hepatitis fields, that single checkpoint inhibition (CPI), such as PD1/PDL1, is not sufficient to fully reinvigorate the exhausted T cell response, has led researchers to look for more effective combination strategies. In this line, the review by the Wedemeyer’s group addresses HCC cures by describing the effectiveness of the association of CPI with angiogenesis inhibition for HCC cures, or with loco-regional treatments that, inducing a massive release of antigens by dying tumor cells, can strongly stimulate immunity. Another intriguing approach to potentiate CPI, thus far tested in pre-clinical and clinical settings of solid cancer, is represented by the oncolytic virotherapy (OV), which relies on viral vectors genetically modified to replicate primarily in tumor tissue to recall intratumor infiltrating lymphocytes and to enhance PDL1 expression on cancer cells [4]. 

Association strategies and other different approaches in NK and T cell-based therapies for HCC have been summarized also in the review by Kalathil and Thanavala, including the preparation of genetically engineered CAR-NK and CAR-T cells, with the first displaying a shorter life-span and thus a reduced risk for autoimmunity induction. In addition, CAR-NK cells do not have any requirement for autologous cells for adoptive transfer [5].

Additionally, in the field of chronic viral hepatitis, it is generally felt that CPI should be associated with a complementary approach for an effective immunotherapy. In this line the manuscript by Pena-Asensio et al. analyzes the possible combinations of PD1 blockade with gamma-chain receptor cytokines to restore virus-specific CD8 T cells in chronic HCV infection. IL7, IL15, and IL21 seem to be good candidates for the homeostatic maintenance of T cell memory, T cell exhaustion decrease and also metabolic reprogramming. Indeed, at least the synergic effect of anti-PDL1 and IL7 has been tested in vitro, showing to be effective on HCV-specific CD8 cell reactivity. Analogously, IL15 or IL21 combined with PD1 blockade display the potential for exhausted CD8 T cell restoration [6].

Cytokine modulation is one of the strategies analyzed also by Barili et al. in the setting of chronic viral hepatitis B and C, among different immunotherapeutic approaches aimed at virus-specific CD8 T cell restoration, such as innate immunity agonism, CPI, metabolic and epigenetic modulation. This review highlights how a deep comprehension of immunopathogenetic mechanisms underlying CD8 T cell exhaustion and linked to stress responses, deregulated metabolism and epigenetics is required for the identification of new targets to be exploited for successful immunotherapy [7].

A focus on CD4 T cells in chronic hepatitis B is the objective of the review paper by Buschow and Jansen, that highlights the importance of targeting also this arm of the T cell response, in addition to CD8 T cells that are more often considered in the immunotherapeutic strategies designs. As CD4 T cells can also be dysfunctional and result in overexpression of co-inhibitory receptors, the combination of therapeutic vaccination with CPI or with metabolic modulators could be successful to restore anti-HBV T cell reactivity, provided that HBeAg levels are reduced to avoid Th2 skewing. Of course, timing for the succession of different combined interventions needs to be well calibrated: first the viral antigenic burden, including HBeAg, should be reduced by antiviral treatments, then therapeutic vaccination could be considered associated with a Th1 skewing adjuvant, before or after T cell-enhancing drugs. Finally, antiviral therapy suspension can also function as a natural booster, eventually resulting in infected hepatocyte clearance [8]. 

A different approach for T cell-based immunotherapy is illustrated in the commentary by Hafezi et al., where adoptive cell therapy with autologous engineered T cells is considered in case of HBV-related HCC recurrence following liver transplantation. As these patients undergo immunosuppressive treatments that can compromise T cell reactivity, a new class of HBV-specific TCR-redirected T cells can be made temporarily resistant to immunosuppression, thanks to mutations in drug-specific target proteins, thus opening up new ways not only in case of HCC liver transplantation, but also for patients with other pathologies that need immunosuppressant therapies [9].
